# Herbal versus synthetic drugs; beliefs and facts

**Published:** 2015-01-01

**Authors:** Ali Karimi, Maedeh Majlesi, Mahmoud Rafieian-Kopaei

**Affiliations:** ^1^Medical Plants Research Center, Shahrekord University of Medical Sciences, Shahrekord, Iran; ^2^Faculty of Nursery and Midwifery, Iran University of Medical Sciences, Tehran, Iran

**Keywords:** Herbal medicine Medicinal plants, Synthetic drugs, Oxicity, Holistic therapy

## Abstract

Herbal therapy is a holistic therapy, integrating emotional, mental and spiritual levels. Life style, emotional, mental and spiritual considerations are part of any naturopathic approach. The use of herbs does not generally involve "drug" actions or adverse effects. Although medicinal plants are widely used and assumed to be safe, however, they can potentially be toxic. Where poisoning from medicinal plants has been reported, it usually has been due to misidentification of the plants in the form, in which they are sold, or incorrectly preparation and administration by inadequately trained personnel. There are some "drug like" plants remedies that their actions approach that of pharmaceuticals. Herbalists use these plants in treatment strategies and in countries such as Britain their vast availability is restricted by law. Digitalis is one of these examples and the number of these plants is not a lot. The mechanisms by which the herbs generally act are not established, however, most of medicinal plants possess antioxidant activities. The plants have been shown to effective by this property is various conditions including cancer, memory deficit and Alzheimer, atherosclerosis, diabetes and other cardiovascular diseases. Antioxidant activities of herbal medicines are also effective in reducing the toxicities of toxic agents or other drugs.

**Implication for health policy/practice/research/medical education:**

Herbal therapy is a holistic therapy, integrating emotional, mental and spiritual levels. Life style, emotional, mental and spiritual considerations are part of any naturopathic approach. The use of herbs does not generally involve "drug" actions or adverse effects. Although medicinal plants are widely used and assumed to be safe, however, they can potentially be toxic. Where poisoning from medicinal plants has been reported, it usually has been due to misidentification of the plants in the form, in which they are sold, or incorrectly preparation and administration by inadequately trained personnel.

About 8% of hospital admissions in the United States of America are due to adverse or side effects of synthetic drugs (1). Approximately 100,000 people each year die due to these toxicities. It means that the killed people in the U.S. by pharmaceutical drugs are at least three times more than the killed by drunken drivers. Each year also thousands of people die from supposedly “safe” over-the-counter drugs. Deaths or hospitalizations due to herbs are so rare that they are hard to find. Even, the National Poison Control Centres of the United States does not have a category in their database for side or adverse reactions to herbs (1,2). Therefore, people every year turn to herbal medicine because they believe plant remedies are free from undesirable side effects (1-5). However, toxicity of herbal medicines needs to be seen in context (2-5). Although herbal medicines are generally considered to be safe and effective, but conventionally it is said that if a drug is effective, it would have side effects. Hence, herbal remedies as drugs either have adverse effects or are not effective (2-5).

There are some “drug like” plants remedies that their actions approach that of pharmaceuticals. Herbalists use these plants in treatment strategies and in countries such as Britain their vast availability is restricted by law. Digitalis is one of these examples and the number of these plants is not a lot (2-6).

The mechanisms by which the herbs generally act are not established, however, most of medicinal plants possess antioxidant activities (3-9). The plants have been shown to effective by this property is various conditions including cancer (10-12), memory deficit and Alzheimer (13,14), atherosclerosis (15-17), diabetes (18-20) and other cardiovascular diseases (21,22). Antioxidant activities of herbal medicines are also effective in reducing the toxicities of toxic agents (23-25) or other drugs (25-27).

The herbal medicines contain a lot of different compounds which some of them have great complexities. Plants substances such as polysaccharides, mucilages and tannins may modulate and modify the effects of “active components”. It has been shown that the whole plants extracts cannot be mimicked by administering purified and isolated constituents of the herbs (27,28).

The sciences believe that the whole plant is greater than the sum of the parts which reflects the inherent conservatism of the medical establishment (1-4).

In this regard, a pharmaceutical drug is usually designed to elicit a specific reaction and its “side or adverse effects” are usually traded as a “risk” against the “benefit” of the primary effect. Herbal medicines usually tend to have several broad complementary or synergistic actions on physiological systems at the same time which are usually in the same general therapeutic direction, and often non-specific. Furthermore, these actions are rarely adverse effects. Herbal medicine actions are too complex and usually cannot be adequately described using the vocabulary of “medication” action terms such as diuretic (2-7).

Herbal therapy is a holistic therapy, integrating emotional, mental and spiritual levels. Life style, emotional, mental and spiritual considerations are part of any naturopathic approach. The use of herbs does not generally involve “drug” actions or adverse effects. Of course, informed knowledge of the effects of medicinal plants as well as doing a clinical trial to understand the appropriate medical application is necessary. It has been suggested that we use the terms indications and contraindications for using a herb instead of “side effects” (2-7).

Synthetic drugs address symptoms caused by specific diseases as understood by scientific pathology, however, a herbal medicine usually direct towards aiding the body’s own healing process. Herbal medicines usually act gently, “suppor” the systems and processes that have become deficient or attempt to help remove excesses that have become preponderant. Symptom relief is only a section of medicinal plants therapeutic strategies. For example, arthritic is usually treated with steroid anti-inflammatory drugs which have widespread disturbing adverse effects. The approach of herbs to these conditions causes moistening of dry synovia, stimulation of circulation in the affected regions, facilitation of elimination via kidneys and hepatic/biliary routes, dietary modification of metabolism, etc. (1-5).

Many people who seek medicinal plants have already been involved in pharmaceutical therapies. Medicinal plants may act as agonists or potentiate some other drugs. Herbalists usually do not like to treat the primary presenting symptom undergoing drug treatment but rather concentrate on supporting other systems and functions stressed by the primary symptom. This allows the body to recover its strength and healing potential so it can then direct these capabilities toward repairing the presenting condition (1-5,24-27).

Ordinary foods may also contain substances such as alkaloids of the Solanaceae, the cyanogenic glycosides in many fruit seeds, the thiocyanates of the brassica vegetables, alpha gliadin produced by gluten in wheat oats and rye, lectins of many pulses including soya and red kidney beans that can be regarded as potentially poisonous. However, these foods are generally regarded as safe. Similarly, both water and oxygen can kill in excessive amounts, so quantity is often an important consideration (2-4).

In practice, three groups of herbs can be identified from a safety point of view (23-30). 1) The herbs that contain near pharmaceutical concentrations of poisonous constituents which should not be taken internally by unqualified persons. Examples are Aconitum spp, Arnica spp, Atropa belladonna and Digitalis spp. 2) The herbs with powerful action and safe under appropriate conditions. 3) Finally, The group of herbs which have been alleged to exhibit specific kinds of toxicities. The best known are Dryopteris, Viscum, and Corynanthe and the hepatotoxicity of pyrrolizidine-alkaloid containing plants such as Comfrey.

Pregnancy is particular situation in which minimal medical intervention should be considered, and some suggest that pregnancy should be considered as “contraindication” to take any medication unless the established safe ones. The question regarding the use of medicinal plants in pregnancy has not been answered except for limited ones. The evidence of teratogenicity in humans arising from herbal remedies is rare and hard to come by. Therefore, it is better to be avoided during pregnancy (23-30).

Herbalists suggest that scientific studies with isolated compounds, in vitro studies, on non-human or even non mammalian organisms, with doses tens or hundreds times of the equivalent medicinal doses, have no arguable extrapolation to the clinical situation using whole herb at appropriate medicinal doses (21-30).

Lack of herbal knowledge by some scientific investigators may lead to misleading results. The commonest mistakes in different trials are the failure to verify the actual identity of plant material used in their experiments and the detection of contaminants. The double blind placebo controlled clinical trial is open to a range of criticisms from the paradigm employed by herbalists is another story (23-30).

In sum it is suggested that the majority of medical plants are safe for non-pregnancy consumption; however, the follow simple but sensible guidelines should be considered in self-treatment:

New or unproven remedies should be avoided.

Only herbs recommended in respected herb books should be used.

The same as synthetic drugs, drug interactions and contraindications must be considered on an individual basis.

The same as synthetic drugs, the herb consumption usually need to be discontinued if adverse reactions took place.

Patients or physicians should not engage in herbal usage for complex conditions without knowledge.

Finally, it is better to avoid herbal remedies during pregnancy.

In overall, herbal therapy is a holistic therapy, integrating emotional, mental and spiritual levels. Life style, emotional, mental and spiritual considerations are part of any naturopathic approach. The use of herbs does not generally involve “drug” actions or adverse effects. Although medicinal plants are widely used and assumed to be safe, however, they can potentially be toxic (31-36). Where poisoning from medicinal plants has been reported, it usually has been due to misidentification of the plants in the form, in which they are sold, or incorrectly preparation and administration by inadequately trained personnel.

## Author’s Contribution

All authors contributed equally to prepare the manuscript and confirmed its final proof.

## Ethical considerations

Ethical issues (including plagiarism, misconduct, data fabrication, falsification, double publication or submission, redundancy) have been completely observed by the authors.

## Conflict of interests

None to declare.

## Funding/Support

Thanks to Shahrekord University of Medical Sciences for its support.
